
JOURNAL INFORMATION
==============================
NLM Title Abbreviation: Curr Med Sci
Journal ID: Curr Med Sci
NLM Journal ID: 101729993

Current Medical Science

ISSN: 2096-5230
EISSN: 2523-899X

ARTICLE INFORMATION
==============================
PMCID: PMC7902569
PMID: 32681265
DOI: 10.1007/s11596-020-2218-9
Article ID: 2218
Article version: 1
Subjects: Erratum

Erratum to: Kinetic Characterization of Tyrosinase-catalyzed Oxidation of Four Polyphenols

Liu Wan-yu m201875230@hust.edu.cn
1
Zou Cong-ming zoucongmingzcm@163.com
2
Hu Jian-hua 1
Xu Zi-jun 1
Si Lu-qin 1
Liu Jun-jun junjun.liu@hust.edu.cn
1
Huang Jian-geng jiangenghuang@hust.edu.cn
1
1 grid.33199.31 0000 0004 0368 7223 School of Pharmacy, Tongji Medical College, Huazhong University of Science and Technology, Wuhan, 430030 China
2 grid.410732.3 0000 0004 1799 1111 Yunnan Academy of Tobacco Agricultural Sciences, Kunming, 650021 China
Electronic publication date: 2020 Jul 17
Print publication date: 2020
Volume: 40
Issue: 3
First page: 594
Last page: 594
Copyright: © The Author(s) 2020
License: Open Access This article is licensed under a Creative Commons Attribution 4.0 International License https://creativecommons.org/licenses/by/4.0/), which permits use, sharing, adaptation, distribution and reproduction in any medium or format, as long as you give appropriate credit to the original author(s) and the source, provide a link to the Creative Commons license, and indicate if changes were made.
License URL: https://creativecommons.org/licenses/by/4.0/

Erratum for: Kinetic Characterization of Tyrosinase-catalyzed Oxidation of Four Polyphenols. 40 2 26 4 2020 239 248 Curr Med Sci 10.1007/s11596-020-2186-0 32337685
issue-copyright-statement: © Huazhong University of Science and Technology 2020

==============================
The online version of the original article can be found at 10.1007/s11596-020-2186-0

